# The etiology of acute meningitis and encephalitis syndromes in a sentinel pediatric hospital, Shenzhen, China

**DOI:** 10.1186/s12879-019-4162-5

**Published:** 2019-06-26

**Authors:** Hongwei Shen, Chunqing Zhu, Xiaorong Liu, Dongli Ma, Chunli Song, Lintao Zhou, Zuer Wang, Yongxuan Ou, Wen Ma, Xianghui Shi, Xuejun Ma, Yiwen Zhou

**Affiliations:** 1 Shenzhen Hospital of Southern Medical University, Xinhu Rd. 1333, Bao’an District, Shenzhen, 518110 Guangdong China; 2 Futian District Center for Disease Control and Prevention, Hongli Xi lu 8043, Futian District, Shenzhen, 518040 Guangdong China; 3 Shenzhen Children’s Hospital, Shenzhen, China; 4 Guangdong Medical College, Dongguan, China; 5 Chinese Center for Disease Control and Prevention, National Institute for Viral Disease Control and Prevention, Changbai Rd. 155, Changping District, Beijing, 102206 China

**Keywords:** Etiology, Childhood, Meningitis, Encephalitis

## Abstract

**Background:**

Acute meningitis and encephalitis syndromes (AMES) is a severe neurological infection which causes high case fatality and severe sequelae in children. To determine the etiology of childhood AMES in Shenzhen, a hospital-based study was undertaken.

**Methods:**

A total of 240 cerebrospinal fluid (CSF) samples from 171 children meeting the case definition were included and screened for 12 common causative organisms. The clinical data and conventional testing results were collected and analyzed. Whole genome sequencing was performed on a *Neisseria meningitidis* isolate.

**Results:**

A pathogen was found in 85 (49.7%) cases; Group B *Streptococcus* (GBS) was detected in 17 cases, *Escherichia coli* in 15, *Streptococcus pneumoniae* in 14, enterovirus (EV) in 13, herpes simplex virus (HSV) in 3, *N. meningitidis* in 1, *Haemophilus influenzae* in 1, and others in 23. Notably, HSV was found after 43 days of treatment. Twelve GBS and 6 *E. coli* meningitis were found in neonates aged less than 1 month; 13 pneumococcal meningitis in children aged > 3 months; and 12 EV infections in children aged > 1 year old. The multilocus sequence typing of serogroup B *N. meningitidis* isolate was ST-3200/CC4821. High resistance rate to tetracycline (75%), penicillin (75%), and trimethoprim/sulfamethoxazole (75%) was found in 4 of *S. pneumoniae* isolates; clindamycin (100%) and tetracycline (100%) in 9 of GBS; and ampicillin (75%) and trimethoprim/sulfamethoxazole (67%) in 12 of *E. coli*.

**Conclusions:**

The prevalence of *N. meningitidis* and JEV was very low and the cases of childhood AMES were mainly caused by other pathogens. GBS and *E. coli* were the main causative organisms in neonates, while *S. pneumoniae* and EV were mainly found in older children. HSV could be persistently found in the CSF samples despite of the treatment. A better prevention strategy for GBS, the introduction of pneumococcal vaccine, and incorporation of PCR methods were recommended.

## Background

Acute meningitis and encephalitis syndromes (AMES) is a severe neurological infection which causes high case fatality and severe sequelae including hearing loss and cognitive deficit [1, 2]. Children under the age of 5 years old are the main group at risk and the young infants below 2 months old have the highest incidence [2, 3].

A broad range of organisms are responsible for bacterial meningitis, including *Streptococcus pneumoniae*, *Haemophilus influenzae*, Group B *Streptococcus* (GBS), *Neisseria meningitidis*, and *Listeria monocytogenes* [2]. Other causative organisms such as herpes simplex virus (HSV), enterovirus (EV), and Varicella zoster virus (VZV) are associated with aseptic meningitis and encephalitis [4].

*N. meningitidis* and Japanese encephalitis virus (JEV) are the only two causative agents included in the National Infectious Diseases Surveillance System in China. However, due to the nationwide vaccination program of serogroup A and serogroup A plus C meningococcal, and JEV vaccines, the incidence of AMES caused by *N. meningitidis* and JEV has decreased markedly [5]. The annual incidence of Japanese encephalitis (JE) declined from 0.9489/100,000 in 2000 to 0.12/100,000 in 2011 [6, 7]. The annual incidence of meningococcal meningitis declined from 0.18/100,000 in 2005 to 0.02/100,000 in 2010 and a switch of prevalent serogroups was observed after integration of meningococcal polysaccharide vaccine into the national Expanded Program on Immunization (EPI) in 2008 [8, 9]. However, little is known about the frequency of other causative pathogens or the disease burden of AMES in China.

A large population-based surveillance for bacterial meningitis during 2006 and 2009 was undertaken in four cities and it estimated the annual incidence (per 100,000 population) of probable bacterial meningitis in the children < 5 years old ranged from 6.95 to 22.30 [3]. A pathogen was identified in a small proportion of tested cases (2.2%) and the cause of other cases remained undefined [3]. A relative low detection rate (22.6%) was also observed in another Chinese study in which serological assays were used for testing viruses [10].

PCR had great advantage over conventional methods and the yield for detection of bacterial pathogens increased by 20 to 85% in a study conducted in Brazil [11], and it was widely used for detecting the causative pathogens of AMES [11, 12].

Due to the large geographical and economic variation in China, the prevalence of vector-borne diseases such as JE [5], immunization rate, and healthcare quality varied in different regions. Apart from the free vaccines, the coverage of others including pneumococcal and *H. influenzae* type b vaccine would be higher in the developed cities than that in other regions. As a metropolis with over 12 million population and China’s open door to the world, it is urgent to understand the etiology of AMES in Shenzhen, and then provide basis for introducing effective policies for immunization and prevention. Here we undertook a prospective study in a sentinel pediatric hospital which covered 426,000 outpatients and 77,000 inpatients annually to determine the etiology of childhood AMES in Shenzhen and extensive testing was conducted.

## Methods

### Study design

Shenzhen Children’s Hospital (SCH) was chosen as the sentinel hospital. It serves as a referral center in the field of pediatric diseases and treats over 60% of childhood AMES cases in Shenzhen each year.

**Fig. 1:**
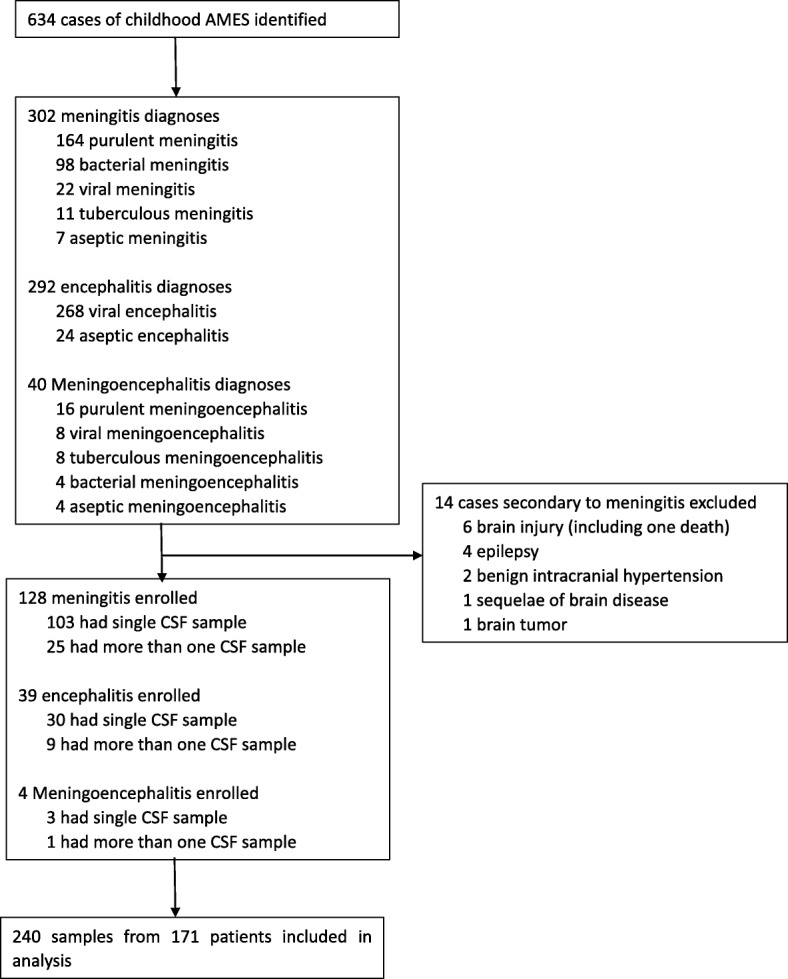
Study profile

Possible AMES were defined based on clinical manifestations (altered consciousness that persisted for longer than 24 h), and with two or more of the following: fever (≥38°C) or the history of fever during the presenting illness; abnormal neuroimaging findings; and CSF pleocytosis (more than four white blood cells per μL). Childhood outpatients and inpatients who met the criteria, including those transferred from other centers in this region were recruited during June, 2015 and October, 2018. Only the possible cases whose guardians agreed to participate in this study and who had enough volume of CSF samples for tests were enrolled in this study. Patients with a different final diagnosis (e.g., tumor and epilepsy) were excluded (Fig. 1).

### Sample collection

Of the enrolled patients, their CSF samples after the routine tests were collected, stored in − 80 °C and transported to Futian District Center for Disease Control and Prevention for PCR examination once every 5 workdays. The demographic data, blood and CSF test results including Gram stain and culture results were collected. The use of antimicrobials when collecting CSF samples, route of admission, clinical symptoms, treatment and outcome were also collected.

### Conventional testing

The isolates from blood and CSF samples were collected and subjected to antimicrobial susceptibility testing using Vitek 2 Compact system (bioMerieux, France). Reference strains were used as controls according to the guideline. Disc diffusion method was used for testing the antibiotic susceptibility for *N. meningitidis* according to the Clinical and Laboratory Standards Institute (CLSI, 2016) M100 guideline. *S. pneumoniae* (ATCC 49619) and *E. coli* (ATCC 25922) were used as controls for disk diffusion method. The susceptibilities for the tested antimicrobials were classified as resistant, intermediate, and susceptible according to the guideline (CLSI, 2016). *S. pneumoniae* antigen testing was performed on the CSF samples collected from the suspected pneumococcal meningitis using lateral flow assay (Alere, USA). The isolated *N. meningitidis* was serotyped with a commercially kit according to the instruction (Remel, UK).

### PCR amplification

The nucleic acid was extracted using MagNA Pure 96 System (Roche) and followed by detection of 12 pathogens using Real-time PCR assays. The oligonucleotide primers and probes were listed in Table 1. *S. pneumoniae*, *H. influenzae*, GBS, *E. coli*, *N. meningitidis*, and *L. monocytogenes* were detected using GoTaq Probe qPCR Master Mix (Promega, USA). The final concentration for each forward primer, reverse primer, and probe was 0.2 μM, 0.2 μM, and 0.05 μM, respectively. The cycling conditions were as follows: 2 min at 95°C followed by 40 cycles of 15 s at 95°C, 1 min at 60°C. Enterovirus, HSV, JEV, mumps virus (MuV), adenovirus (AdV), and VZV were detected using QuantiTect Probe RT-PCR Kit (Qiagen, Germany) according to the instruction. The final concentration for each forward primer, reverse primer, and probe was 0.4 μM, 0.4 μM, and 0.1 μM, respectively. The cycling conditions included reverse transcription of 30 min at 50°C, a initiating stage of 15 min at 95°C, and followed by 45 cycles of 15 s at 94°C, 1 min at 60°C. The nucleic acids extracted from reference strains, constructed plasmids, or in vitro transcribed RNA templates were used as controls. Amplification, detection, and data analysis were performed in a Bio-Rad CFX96 real-time thermal cycler (Bio-Rad, USA).

**Table 1:** The oligonucleotide primers and probes for real-time PCR used in this study

Organism	Target gene	Primer sequence (5′-3′)	Probe (5′-3′)	Citation
*S. pneumoniae*	*lytA*	F: ACGCAATCTAGCAGATGAAGC R: TGTTTGGTTGGTTATTCGTGC	FAM-TTTGCCGAAAACGCTTGATACAGGG-BHQ	[29]
*H. influenzae*	*bexA*	F: GGCGAAATGGTGCTGGTAA R: GGCCAAGAGATACTCATAGAACGTT	HEX-CACCACTCATCAAACGAATGAGCGTGG- BHQ	[29]
GBS	*cfb*	F: AGCTCTATTAGAAGTACATGCT R: CATTTGCTGGGCTTGATTATT	ROX-ATCAAGTGACAACTCCACAAGTGGTAA- BHQ2	[30]
*E. coli*	*uid*	F: GTGTGATATCTACCCGCTTCGC R: GAGAACGGTTTGTGGTTAATCAGGA	ROX-TCGGCATCCGGTCAGTGGCAGT-BHQ2	[31]
*N. meningitidis*	*ctrA*	F: GCTGCGGTAGGTGGTTCAA R: TTGTCGCGGATTTGCAACTA	FAM- CATTGCCACGTGTCAGCTGCACAT-BHQ	[32]
*L. monocytogenes*	*iap*	F: CTAAAGCGGGAATCTCCCTT R: CCATTGTCTTGCGCGTTAAT	HEX- CTTCTGGCGCACAATACGCTAGCACT- BHQ	[33]
mumps virus	Fusion protein	F:TCTCACCCATAGCAGGGAGTTATAT R: GTTAGACTTCGACAGTTTGCAACAA	HEX-AGGCGATTTGTAGCACTGGATGGAACA- BHQ	[34]
herpes simplex virus	polymerase	F:CATCACCGACCCGGAGAGGGAC R: GGGCCAGGCGCTTGTTGGTGTA	FAM- CCGCCGAACTGAGCAGACACCCGCGC- BHQ	[35]
Japanese encephalitis virus	polyprotein	F: AGAACGGAAGAYAACCATGACTAAA R: CCGCGTTTCAGCATATTGAT	ROX-ACCAGGAGGGCCCGG- BHQ2	[36]
enterovirus	polyprotein	F: CCGGCCCCTGAATGC R: CACCGGATGGCCAATCCA	ROX-AACCGACTACTTTGGGTGTCCGTGTTTC- BHQ2	[37]
adenovirus	*hexon*	F: GCCACGGTGGGGTTTCTAAACTT R: GCCCCAGTGGTCTTACATGCACATC	CY5-TGCACCAGACCCGGGCTCAGGTACTCCGA -BHQ2	[38]
varicella zoster virus	DNA polymerase	F: CGGCATGGCCCGTCTAT R: TCGCGTGCTGCGGC	HEX-ATTCAGCAATGGAAACACACGACGCC- BHQ1	[39]

### Next generation sequencing

The nucleic acids of *N. meningitidis* strains were extracted using QIAamp DNA Mini Kit (Qiagen, Germany) and 1 μg of DNA was broken using ultrasound, concentrated, and used for preparing DNA library using NEB Next® Ultra™ DNA Library Prep Kit for Illumina (NEB, USA). Whole genome sequencing was performed on Illumina Miseq (USA). The sequencing results were edited and assembled using SPAdes and PubMLST was used for identifying alleles for multilocus sequence typing (MLST), antimicrobial resistance related genes including *gyrA*, *penA*, and *rpoB*, and peptide coding gene such as *porA*, *porB*, *fetA*, and *fHbp*.

## Results

### Sample information

A total of 634 possible childhood AMES cases were identified during June, 2015 and October, 2018 and 240 CSF samples collected from 171 patients (male, 116) were included (Fig. 1). Of all the samples, 104 sequential samples were collected from 35 patients. Twelve of samples were collected before the use of antibiotics. Patients ranged in age from 0 to 15 years old and 91 of them aged less than 3 months. Of all the patients, 73 were neonates and 27 were preterm; 103 were hospitalized or transferred from other hospitals. The most common symptoms were fever (137, 80.1%), vomiting (73, 42.7%), and seizures (36, 21.1%). Two recurrent cases were found. Thirteen cases were transferred to other hospitals and the symptoms of all the others improved when discharged from SCH. No death was found.

### Etiology

Overall, a pathogen was found in 85 (49.7%) cases. A bacterial pathogen was found in 68 (39.8%) of cases (GBS, 17; *E. coli*, 15; *S. pneumoniae*, 14; *H. influenzae*, 1; *N. meningitidis*, 1; others, 23). Of the other bacteria, *Staphylococcus epidermidis* was the predominant microorganism (9 cases) and all were isolated from patients during hospitalization and after administration of antibiotics. EV and HSV were found in 13 and 3 cases of viral infections, respectively. *Candida famata* was isolated from the blood sample from a neonate (Table 2). No *L. monocytogenes*, JEV, MuV, AdV, and VZV were detected.

**Table 2:** Pathogens detected in the 171 AMES cases by culture/PCR method

Organism^a^	Blood	CSF			Total^c^
		culture	PCR	LFA^b^	
*S. pneumoniae* ^d^	7	9	3	11	14
*E. coli* ^e^	12	8	2	–	15
GBS^f^	13	5	4	–	17
*H. influenzae*	0	0	1	–	1
*N. meningitidis*	1	0	1	–	1
Others^g^	14	9	–	–	23
enterovirus	–	–	13	–	13
HSV ^h^	–	–	3	–	3
total	47	31	27	11	85^i^

-, not detected

^a^ GBS, group B *Streptococcus*; HSV, herpes simplex virus; JEV, Japanese encephalitis virus; VZV, varicella zoster virus

^b^ LFA, lateral flow assay

^c^ A causative agent was defined by a positive result tested by any of the methods

^d^ *S. pneumoniae* was isolated from both blood and CSF samples in 7 cases. Three pneumococcal cases were detected only by LAF due to the use of antibiotics before sampling

^e^ *E. coli* was isolated from both blood and CSF samples in 5 cases

^f^ GBS was isolated from both blood and CSF samples in 4 cases

^g^ Others included 6 of *Staphylococcus epidermidis*, 2 of *Pseudomonas aeruginosa*, and 1 *Staphylococcus capitis* isolate from CSF samples; and 3 *Staphylococcus epidermidis,* 4 *Klebsiella pneumoniae*, 1 *Staphylococcus capitis*, 2 *Staphylococcus hominis*, 1 *Acinetobacter baumannii*, 1 *Staphylococcus aureus*, 1 *Candida famata*, and 1 *Enterococcus aerogenes* isolate from blood samples

^h^ The 3 isolates were typed using a commercial kit (Daan, China) and all were identified as HSV-1

^i^ Recurrent and co-infection were excluded

GBS and *Pseudomonas aeruginosa* was isolated from a long-hospitalized case in the 1st and 9th month, respectively. Of the recurrent cases, *S. pneumoniae* was found in 2 episodes of meningitis in case 1. *S. pneumoniae* and *E. coli* was found in 1st and 2nd episode of meningitis in case 2, respectively.

### Age distribution of the main pathogens

GBS and *E. coli* were the predominant causes of bacterial meningitis in neonates. Twelve of 17 GBS and 6 of 15 *E. coli* meningitis were found in neonates aged less than 1 month. All the pneumococcal meningitis was detected in patients more than 3 months old and 13 of 14 cases was found in the age group of 3 months and 5 years old. Of the 13 cases of encephalitis caused by EV, 12 was found in children over 1 years old and 8 over 5 years old. Two of 3 HSV encephalitis cases were 2 months old.

### CSF examination in AMES caused by common pathogens

The mean white blood cell (WBC) count (10^6^/μl), protein, and glucose in CSF, and hospitalization length in EV encephalitis was 143, 0.40 g/L, 3.23 mmol/L, and 7 days, respectively. A higher mean CSF WBC count and protein, longer hospitalization days, and lower CSF glucose were seen in the bacterial meningitis cases. A positive PCR result for HSV could be found in the CSF sample after 43 days of treatment. The longest period after treatment to obtain a positive PCR result for *E. coli*, *N. meningitidis, S. pneumoniae,* and GBS was 13, 8, 17, and 9 days, respectively.

### Antimicrobial susceptibility profiles of the main microorganisms

Of the 4 tested isolates of *S. pneumoniae*, three were resistant to tetracycline, penicillin, and trimethoprim/sulfamethoxazole. All the 9 GBS isolates were resistant to clindamycin and tetracycline. Of the 4 GBS strains whose resistance to roxithromycine, azithromycin, and clarithromycin was tested, they were all resistant to these drugs (not shown). The highest resistance rate in *E. coli* was found in ampicillin (9/12), followed by trimethoprim/sulfamethoxazole (8/12). Resistance to penicillin, and oxacillin was found in all the 8 tested *S. epidermidis* isolates (Table 3).

**Table 3:** The antimicrobial resistance profile of the main microorganisms isolated from childhood AMES

Antibiotics	Microorganisms							
	*S. pneumoniae* (*n* = 4)		*S. epidermidis* (*n* = 8)		GBS (*n* = 9)		*E. coli* (*n* = 12)	
	MIC range (μg/ml)	No. of resistant strains	MIC range (μg/ml)	No. of resistant strains	MIC range (μg/ml)	No. of resistant strains	MIC range (μg/ml)	No. of resistant strains
Penicillin								
amoxicillin	≤ 1	0	–	–	–	–	–	–
ampicillin	–	–	–	–	≤ 0.25	0	≤ 2- ≥ 32	9
oxacillin	–	–	≥ 4	8	–	–	–	–
penicillin	≤ 0.06- ≥ 2	3	≥ 0.5	8	≤ 0.12	0		–
Cephalosporins								
cefazolin	–	–	–	–	–	–	≤ 4- ≥ 64	6
cefepime	–	–	–	–	–	–	≤ 1- ≥ 64	2
ceftazidime	–	–	–	–	–	–	≤ 1–16	3
ceftriaxone	≤ 1	0	–	–	–	–	≤ 1- ≥ 64	6
Glycopeptide								
vancomycin	–	–	≤ 2	0	≤ 0.5	0	–	–
Aminoglycoside								
gentamicin	–	–	≤ 0.5	0	–	–	≤ 1- ≥ 16	4
Tetracycline								
tetracycline	4- ≥ 16	3	≤ 2	0	≥ 16	9	–	–
Fluoroquinolone								
ciprofloxacin	–	–	≤ 2	0	≤ 0.5	0	≤ 0.25- ≥ 4	5
levofloxacin	≤ 1	0	≤ 2	0	≤ 1	0	≤ 0.25- ≥ 8	5
Lincosamide								
clindamycin	–	–	≤ 0.25- ≥ 8	3	≥ 0.5	9	–	–
Folate pathway inhibitor								
trimethoprim/sulfamethoxazole	20–80	3	≤ 10- ≥ 320	5	–	–	≤ 20- ≥ 320	8
β-lactam/β-lactamase inhibitor								
ampicillin/sulbactam	–	–	–	–	–	–	≤ 2- ≥ 32	6
Monobactam								
aztreonam	–	–	–	–	–	–	≤ 1- ≥ 64	3
Carbapenem								
imipenem	–	–	–	–	–	–	≤ 1	0

*MIC* minimum inhibitory concentration

- not tested

### Characterization of *N. meningitidis*

A serogroup B *N. meningitidis* was isolated from the blood sample of a 3-month-old meningococcal case. It was also detected in the CSF samples by PCR (Table 2). The isolate was sensitive to meropenem, ceftriaxone, chloramphenicol, and cefotaxime, and intermediate sensitive to penicillin. The whole genome was 2,305,805 bp and the MLST type was ST-3200/CC4821. The allele for the antibiotic-associated gene *gyrA*, *penA*, and *rpoB* was 116, 1, and 85, respectively. The allele for peptide coding gene *porA*, *porB*, *fetA*, and *fHbp* was 20,14; 34; F3–9; and 16/A19, respectively.

## Discussion

A pathogen was identified in 85 (49.7%) cases and their age distribution and prevalence of antibiotic resistance were elucidated. These results could provide basis for designing prevention policy, as well as for the diagnosis and empirical treatment of AMES. Therefore, to enable the timely and appropriate treatment and to improve the outcome.

Compared with 2.2% in a large surveillance study in which only the laboratory confirmed *S. pneumoniae*, *H. influenzae* type b, and *N. meningitidis* isolates were included [3], the detection rate in our study was greatly improved. In another study where serological tests were used for detecting viruses, a causative agent was found in 22.6% (538/2382) of cases [10]. The use of PCR method and childhood population might contribute to the relative higher detection rate in this study. The absence of JEV, MuV, and VZV might due to the difference in sample size, regional or demographic factors. The changes of immunization policies might also affect the results, such as the low detection rate of JEV.

Despite of the usefulness of conventional methods, incorporation of PCR method could improve the outcome of surveillance. In the culture negative samples, additional 13 EV, 3 HSV, 2 GBS, and 1 *H. influenzae* were identified. The incidence of *H. influenzae* meningitis was very low due to the use of Hib vaccine in Shenzhen and it would not be considered without a positive result. The positive PCR result could help initiate timely and appropriate therapy in the clinical setting. However, due to the long course of AMES and early initiation of empirical treatment, 228 of 240 CSF samples were collected after the use of antibiotics. The presence of antibiotics in CSF might shorten the time for the detection of pathogens by PCR. In the sequential samples, the negative results were obtained after administrating antibiotics for a period of time, which was also observed in another study [13].

Due to the nationwide vaccination of meningococcal and JEV vaccines, the annual incidence of meningococcal disease and JE in Shenzhen decreased to 0.048 and 0.664 (per 100,000), respectively [14, 15]. The absence of JE in this study could be attributed to its low incidence. The detection of serogroup B *N. meningitidis* was attributed to the group specificity of meningococcal polysaccharide vaccine and its low cross-immunity rate. The introduction of meningococcal A vaccine in 2000 and meningococcal A plus C polysaccharide vaccine in 2008 in Guangdong has led to serogroup replacement of *N. meningitidis.* During 2008 and 2010, serogroup C meningococcal diseases (MenC) accounted for majority of meningococcal cases, and serogroup B meningococcal diseases (MenB) occurred relatively infrequently. From 2011 onwards, the proportion of MenC cases began to decline and MenB to rise [8, 16, 17]. Other strains with the same MLST type were isolated in France, UK, and Taiwan, which caused meningococcal disease mainly in young children aged less than 1 years old [18].

GBS was one of the predominant causes of bacterial meningitis in infants<3 months, despite of the high coverage of prenatal screening in Shenzhen. The high incidence of GBS meningitis in this age group was also observed in the UK [19], which was unchanged from a decade ago [20], suggesting the poor impact of the prevention strategy. Therefore, a better policy for prevention is urgent. The GBS isolates were susceptible to most antibiotics, but showed a high resistance rate to clindamycin and tetracycline, which was consistent with other study [21]. The antimicrobial susceptibility profile should be considered in the empirical treatment of GBS meningitis.

Coagulase-negative Staphylococcus (CoNS), mainly *S. epidermidis* was one of the most commonly isolated bacteria. However, it was excluded in other studies due to its colonization on the skin surface [22]. A contamination was not able to be discarded in the 13 CoNS isolates from CSF or blood samples. However, all the 9 *S. epidermidis* strains were isolated from hospitalized cases after the use of antibiotics, suggesting the possibility of nosocomial infections. The high incidence and occurrence of multi-drug resistance strain posed the necessity for designing effective prevention strategies.

*S. pneumoniae* was the leading cause of bacterial meningitis in the group aged between 3 months and 5 years. The estimated annual incidence of pneumococcal meningitis was 14 per 100,000 in children between 1 month and 59 months in China [23]. According to another research, the most common serogroups were 19F, 19A, and 14, which were covered by the 13-vulent pneumococcal conjugate vaccine (PCV13) [23, 24]. The introduction of PCV13 has resulted in significant reductions of pneumococcal meningitis caused by new serotypes [25]. But PCV13 was not included in the National Planned Immunization schedule and the vaccination rate was unknown. To reduce the incidence of pneumococcal meningitis, the introduction of PCV13 vaccine and further long surveillance were needed. The high resistance rate to tetracycline, penicillin, and trimethoprim/sulfamethoxazole needed the confirmation with large sample size.

In the aseptic meningitis and encephalitis, EV accounted for the majority of viruses detected and 92% of EV infection was found in children over 1 year of age, in agreement with the previous study [26]. No serious complications or deaths were found. EV PCR was not routinely performed for diagnosis of central nervous system (CNS) infections in the sentinel hospital, and there was a delay of diagnosis when the samples were transported to CDC after several days of sampling. Therefore, 11 of 13 EV was found in the samples with unknown cause. As a result, a longer mean duration of hospitalization stay (7 days) and antibiotic administration (6 days) was observed, compared with a previous study (3 and 2 days, respectively) [26]. It was highly recommended to incorporate EV PCR into the routine test for diagnosis of CNS infection in the group at high risk.

Similarly, long mean stay was observed in HSV infections (25 days) and 2 of 3 HSV-positive cases were treated as bacterial meningitis initially. The clinical findings of Herpes simplex encephalitis (HSE) were not pathognomonic and numerous other CNS infections could mimic HSE, so it was important to identify HSV in the high-risk population. All the 3 HSE cases were found in children below 2 years old, consistent with a prior study which indicated the predisposition to HSE was tightly age-dependant [27]. Due to the long duration of disease course, efficacy of acyclovir treatment, high mortality and severe sequelae [28], it was important to identify HSE. Therefore, the appropriate treatment could be given and the outcome could be improved.

This study had several limitations. Firstly, all the samples were collected from one sentinel hospital and the results may not be representative of this region. Secondly, a large proportion of CSF samples subjected to PCR detection were collected after the use of antibiotics, which might result in negative results. Thirdly, a long storage time of CSF samples has led to a delay of diagnosis. Finally, the long-term outcome was not followed and the case fatality might be underestimated. Nevertheless, due to the difficulty in collecting CSF samples from children, this was the most comprehensive data collection on the etiology of AMES in this region. This referral center received childhood AMES cases from many other centers in Shenzhen made the results reasonably representative. A prospective study involved more sentinel hospitals is anticipated to overcome these limitations.

## Conclusion

Due to the nationwide use of serogroup A and serogroup A plus C meningococcal, and JEV vaccines, the incidence of meningococcal disease and JE is very low in Shenzhen. The main causative agents for childhood AMES were GBS, *S. pneumoniae, E. coli*, EV, and HSV. A better prevention strategy for GBS, the introduction of PCV13 vaccine, and incorporation of PCR methods were recommended to reduce the incidence of AMES and achieve early diagnosis.

## Data Availability

The datasets used and analyzed during the current study are available from the corresponding authors on reasonable request.

## Abbreviations

AdV: Adenovirus
AMES: Acute meningitis and encephalitis syndrome
CDC: Center for Disease Control and Prevention
CLSI: Clinical and Laboratory Standards Institute
CNS: Central nervous system
CoNS: Coagulase-negative Staphylococcus
CSF: Cerebrospinal fluid
EPI: Expanded Program on Immunization
EV: Enterovirus
GBS: Group B *Streptococcus*
HSE: Herpes simplex encephalitis
HSV: Herpes simplex virus
JE: Japanese encephalitis
JEV: Japanese encephalitis virus
LFA: Lateral flow assay
MenB: Serogroup B meningococcal diseases
MenC: Serogroup C meningococcal diseases
MIC: Minimum inhibitory concentration
MLST: Multilocus sequence typing
MuV: Mumps virus
PCV13: 13-vulent pneumococcal conjugate vaccine
SCH: Shenzhen Children’s Hospital
VZV: Varicella zoster virus
WBC: White blood cell
